# A novel anchoring system for pelvic organ prolapse repair: an observational study

**DOI:** 10.1007/s00192-022-05444-7

**Published:** 2023-01-16

**Authors:** Heather van Raalte, Nina Bhatia, Jeffrey Mangel, Hugo Ryckebusch, Jan-Paul Roovers

**Affiliations:** 1 Penn Medicine Princeton Medical Center, Princeton, NJ USA; 2 Hackensack Meridian Health Medical Group, Old Bridge, NJ USA; 3 MetroHealth Medical Center, Cleveland, OH USA; 4 Coloplast Manufacturing France, Le Plessis-Robinson cedex, France; 5 Amsterdam UMC, location AMC, Meibergdreef 9, 1105 AZ, Amsterdam, Netherlands; 6 Bergman Clinics Netherlands, Amsterdam, Netherlands

**Keywords:** Anchoring device, Native tissue surgery, Pelvic organ prolapse, Sacrospinous ligament fixation, Safety

## Abstract

**Introduction and hypothesis:**

Sacrospinous ligament (SSL) fixation is an effective and widely used vaginal procedure for correcting apical prolapse. The Saffron Fixation System (Coloplast Corp., Minneapolis, MN, USA) is a new anchoring device aimed at facilitating a durable, easy, and short procedure for SSL fixation with the goal of minimizing operative complications. The objective was to demonstrate the efficacy and safety of anchor deployment and suture fixation for pelvic organ prolapse repair using the Saffron Fixation System.

**Methods:**

An observational human cadaver study was conducted to measure the distance between anchor location and anatomical landmarks in the pelvis, and the holding force of the fixated anchors. Anchors were placed in four human cadavers by different implanters. The pull-out force of these anchors was measured to assess efficacy (three cadavers by three implanters) and the distance between anchors and primal vessels and nerves was measured to assess safety (one cadaver by one implanter).

**Results:**

Nineteen out of 20 anchors (95%) were correctly placed as judged by independent assessment performed by non-implanting surgeons. Distance between anchors and surrounding nerves and vessels exceeded 10 mm. Mean (SD) pull out-force was 17.9 (5.6) N.

**Conclusion:**

The innovative anchoring device that was developed appeared to enable precise and solid anchor placement in the SSL. Future clinical studies are needed to explore if the theoretical advantages of this device translate to improved clinical outcomes in comparison with available suturing and anchoring devices.

## Introduction

Pelvic organ prolapse (POP) is a prevalent condition, with 1 in 10 women requiring surgical correction during their lifetime [1]. Sacrospinous ligament (SSL) fixation intending to optimize apical suspension has become the cornerstone of successful vaginal prolapse surgery [2]. Precise and reliable fixation is vital for good surgical outcomes and several suturing and anchoring devices have been introduced over the years to facilitate precise and reliable fixation [3, 4]. From a safety standpoint, it is critical that fixation devices minimize possible hazard to nerves and vessels (particularly the pudendal nerve and inferior gluteal artery) that are closely related to the SSL [5–7].

Limitations of available fixation devices include suboptimal ergonomics, which may limit precision, larger anchor size, limited suture choice, and risk of anchor dislocation in response to increased abdominal pressure. There is a paucity of evidence demonstrating the clinical efficacy, safety, or superiority of device performance, and there remains a lack of documentation regarding the development and engineering rationale of available devices. Coloplast Corp. (Minneapolis, MN, USA) developed the Saffron™ Fixation System with the intent of generating an anchoring device that overcomes the shortcomings of existing devices. We present the results of human cadaver studies to evaluate the performance of this innovative anchoring device.

## Materials and methods

### Device development rationale

The majority of vaginal POP procedures involve apical suspension [8]. In comparison with vaginal hysterectomy with uterosacral ligament suspension, sacrospinous fixation carries a significantly lower risk of recurrent POP [9]. The SSL can be approached from the posterior or anterior vaginal walls. Lo and colleagues [10] showed that the anatomical result is better with a combined posterior and anterior approach. However, a direct fixation to the SSL by an anterior approach is technically challenging owing to the limited space available to place retractors for proper access and visualization. Therefore, a fixation device that could access the ligament by an anterior or posterior approach would be beneficial to minimize dissection, assist with blind suture or anchor passage through the ligament, and increase fixation precision and safety. A fixation device would also be beneficial during graft use as similar landmarks are used to suspend the vagina to the connective tissue of the pelvis.

Anatomical studies reveal that the SSL has a mean length of 43.0 ± 6.6 mm (mean ±SD) and is in close proximity to the following vulnerable structures [5–7, 11]:

1. The inferior gluteal artery emerges from the infrapiriform foramen above the SSL, courses to infero-laterally leaving the pelvis upon and passes close to the upper border of the lateral half of the SSL.
2. The pudendal artery crosses the ischial spine very close to the SSL posteriorly and enteres into the ischio-anal fossa.
3. The sciatic nerve is approximately 25.1 mm lateral to the ligament where there is minimal risk of injury to this structure [5].

It is advisable to avoid the superior portion of the SSL as there is significant risk of damage to the pre-sacral nerves and vessels, as well as the gluteal veins, which are posterior to the SSL [5]. The pudendal nerve turns dorsolateral to the ischial spine and is positioned behind the lateral part of the SSL. Thus, the location of a suture or anchor during an SSL fixation should be aimed at the lower and medial parts of the SSL.

### Device description

**Fig. 1:**
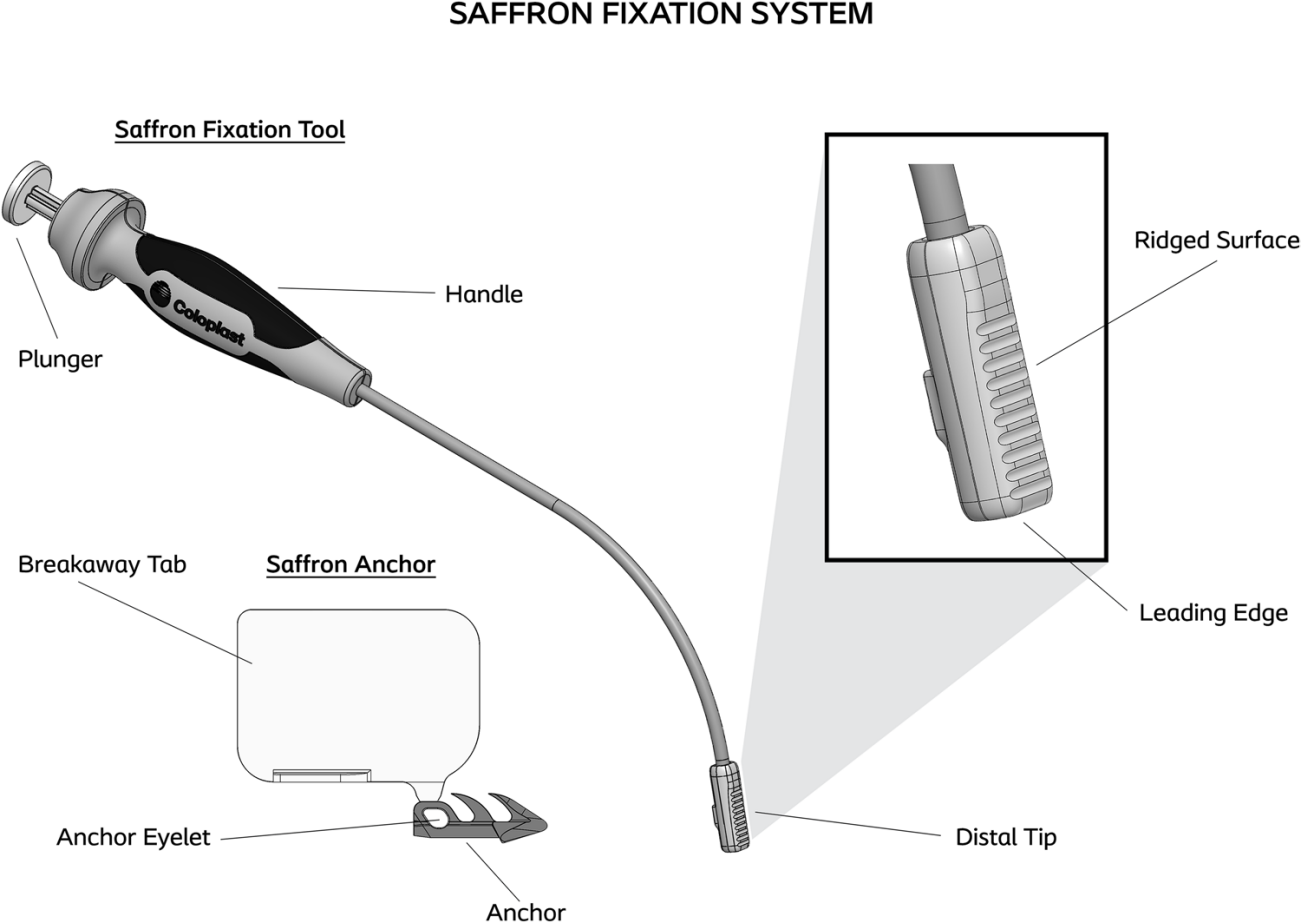
Components of the Saffron device

The Saffron Fixation System consists of the fixation tool and non-absorbable, polysulfone anchors (Fig. 1). This system is intended to create a permanent fixation point in the SSL for suture attachment during vaginal pelvic organ prolapse reconstructive surgery, allowing use of the surgeon’s choice of commercially available off-the-shelf sutures and needles. The fixation tool facilitates anchor placement without direct visualization of the SSL. The anchor is 9.3 mm long and 3.4 mm high. The width of the barbs is 1.3 mm, and the anchor distal end diameter is 1.7 mm. The anchor is loaded in the distal tip of the fixation tool and is deployed by a plunger, which connects the handle and distal tip. The angle of the shaft of the Saffron Fixation Tool makes it possible to visualize the distal tip during introduction and guiding, as it extends almost 7 cm from the middle of the fixation tool handle. The distal tip is 30 cm distal to the plunger. This distance was selected to find an optimal distance between too short (which would prevent the surgeon from visualizing the distal tip) and too long (which would negatively affect the ergonomics of the device).

### Operative technique

First, the Saffron Fixation System is loaded by threading off-the-shelf suture through the anchor eyelet, maintaining control of the suture and anchor with the anchor break-away tab. The anchor sled is aligned with the loading rails of the tool’s distal tip and slid back into the anchor retainers, receiving confirmation of proper loading with a haptic and auditory click. The break-away tab is twisted away like a key and discarded, and the device is ready for anchor deployment.

The index finger is used to guide the distal tip of the Saffron device to the desired fixation point. After proper positioning at the fixation landmark, finger pressure is applied to the ridged surface of the distal tip to provide control and ensure precise deployment. The plunger is firmly depressed until a click is felt, indicating that the anchor has been implanted into tissue. The plunger is released and the tool is withdrawn.

The anchor is palpated to confirm the location, and gentle traction is applied on the suture to ensure adequate fixation. Some amount of anchor eyelet protrusion is considered acceptable.

### Methods

An observational cadaver study was performed to collect data on immediate fixation performance and safety as reflected by:

1. Correct anchor placement identified by palpation and then dissection
2. Distance between anchors and vulnerable structures in the pelvic cavity
3. The pull-out forces required to pull the anchor elements

The researchers determined that studying four samples would be enough to answer the research questions. As no living subjects were evaluated in this study, there was no need to obtain approval of an ethics committee.

The data on continuous variables with normal distribution were presented as mean ± SD and compared in study groups using Student’s *t* test. One-sided *p* value of < 0.05 was considered significant.

### Cadaver study

The study included previously frozen non-embalmed female, disarticulated torso cadavers with no history of prior pelvic surgery for incontinence, pelvic organ prolapse, or pelvic cancer with intact pelvic genitalia. Cadavers 1–3 were used for the efficacy testing (pull-out forces assessment) and cadaver 4 was dedicated to safety testing (measurement of landmark and anchor proximity). Four urogynecological surgeons with extensive experience in performing SSL fixation performed the procedures, and an assistant professor of anatomy performed the safety assessment dissection on cadaver 4. The participating surgeons were trained on the Saffron Fixation System at a previous laboratory and had implanted approximately 10–20 anchors on specimens prior to this cadaver study. The specimens were placed in lithotomy position with the pelvis overhanging the table. For performance and safety testing, the surgeons were asked to insert one anchor into the left and right SSLs consistent with clinical practice; 1 to 2 cm medial to the ischial spine on the anterior half of the ligament via the anterior compartment. All anchors were placed with a 0 Prolene suture to allow identification of its location and pull-out forces measurement. To verify the ability to deliver anchors to the left and right SSLs from both compartments, a second set of anchors were implanted via the posterior compartment for the performance assessment. The anchors placed from the posterior compartment were implanted 1 to 2 cm medial to the first set of anchors placed in the SSLs. Both anterior and posterior surgical approaches were tested for anchor insertion efficacy. To test anchor efficacy, the prolene suture was externalized through the vaginal incision and introitus. The suture was knotted and connected to a dynameter, an instrument for quantifying pullout force. The dynameter was advanced until anchor pullout occurred and the maximum force was registered. As the dynameter was manually pulled, the strain rate was not explicitly controlled (although similar strain rates were attempted by the participants). The axis of pull for the test was parallel to the suture exiting the vagina and not perpendicular to the plane of the SSL.

Assessment of correct anchor location and palpability was conducted by an independent surgeon evaluator who did not implant anchors in a cadaver. Anchor palpability was assessed in the dissected space and through the vaginal wall. To assess anchor palpability through the vaginal wall, the vaginal incision was closed using a clamp to simulate sutured closure. The independent surgeon evaluator attempted to palpate anchors through the vaginal wall using a surgical glove-covered finger. Correct anchor location was assessed by the same evaluator based on predetermined criteria.

## Results

### Efficacy of anchor insertion

For the efficacy evaluation, anchors were placed in cadavers 1–3 by three different implanters. Nineteen out of 20 (95%) anchors were correctly placed, as judged by independent assessment performed by non-implanting surgeons. The force needed to extract the inserted anchors was measured by manual dynamometer. Traction was performed gradually, horizontally parallel to the table with regard to the SSL, and forcefully until the anchor/suture failed. All measurements were repeated and reported in each cadaver. Eighteen anchors were tested for pull-out force at the SSL and the mean extraction force was 17.9 N ± 5.6 N.

### Safety of anchor insertion

During the safety assessment, anchors in cadaver 4 were palpable in the dissected space and no anchors were palpable through the vaginal wall. Some anchors were intentionally placed superficially to assess the impact of incomplete deployment. The superficially placed anchors were not palpable through the vaginal wall and could be removed by gently pulling on the suture and sweeping tissue away from the anchor.

The last specimen pelvis was dissected by a professor of anatomy for validation of the safety of the procedure by identifying the position of the anchors, measuring the distance to the anatomical landmarks, and evaluating for potential injuries to adjacent tissues or organs. After four anchors were inserted, the pelvis was bisected through the medial plane. The symphysis was cut anteriorly, and the coccyx, sacrum, and spine were cut posteriorly. The pelvic organs (bladder, urethra, vagina, uterus, and rectum) were also bisected through the medial plane. The pelvic organs were left intact but detached from the pelvic sidewall to expose the deeper structures within the pelvis. All vessels and nerves to the pelvic organs, including the ureters, were cut at the side wall of the pelvis. Where landmarks were large, nondiscrete structures (e.g., internus obturator muscle, levator ani), pictures were taken instead of measurements of anchor proximity to the landmark. Anchor proximity to some pelvic organs was assessed on structures reapproximated by the anatomist (owing to the organs being bisected). The proximity of some pelvic structures (e.g., uterine artery, ureter) to anchors was not measured owing to large apparent distances from landmark to anchor and disruption of the pelvic organs during bisection.

**Fig. 2:**
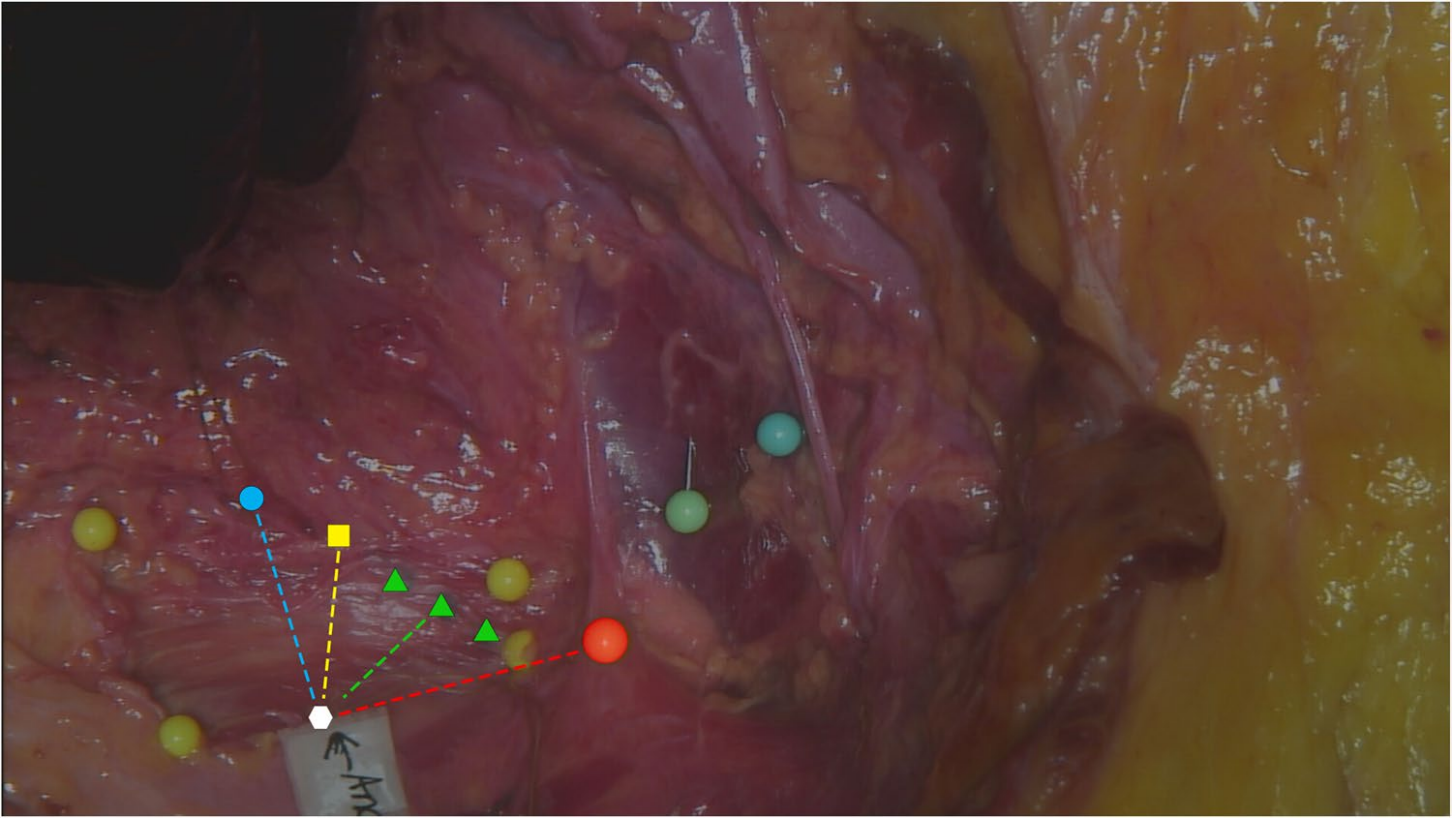
The anatomical position of the Saffron anchor in relation to neighboring tissues and structures. *Blue circle* inferior gluteal artery pelvic exit, *green triangles* pudendal nerve path outside the pelvis (inferior to the sacrospinous ligament), *orange dotted line* pudendal nerve proximity outside the pelvis, *yellow dotted line* ischial spine proximity, *blue dotted line* inferior gluteal artery pelvic exit proximity, *green dotted line* closest distance of the anchor to the pudendal nerve outside of the pelvis, *yellow square* pudendal nerve pelvic exit, *red circle* ischial spine

All coccygeus muscle and loose tissue were removed from the top and bottom of the ligament. Following muscle and tissue removal, the mean thickness of the SSL was found to be 1.5 mm. This removal of all muscle and tissue from the SSL could explain the lower thickness observed in this specimen relative to what other authors have reported. The SSL dimensions were 3.0 cm at the medial border and 1.5 cm at the lateral border. The anchor was placed in the infero-mid part of the edge of the ligament at a mean distance of 1.2 cm from the pudendal nerve (at the closest point outside the pelvis; orange dotted line), 1.7 cm from the pudendal nerve (at the pelvis exit; green dotted line, 2.35 cm from the ischial spine (yellow dotted line), 2.45 cm from the rectum, and 2.9 cm from the inferior gluteal artery (blue dotted line), which coursed laterally toward the superior border of the SSL. We found no damage to the surrounding anatomical landmarks, pelvic organs (bladder and rectal wall), or tissues, with no instances of anchor protrusion through the posterior aspect of the ligament (Fig. 2). The distances between the anchors and the anatomical landmarks are reported in Table 1.

**Table 1:** Mean distance (in centimeters) from the anchors to the anatomical landmarks

	Left sacrospinous ligament	Right sacrospinous ligament
Ischial spine	3.0	1.7
Sciatic nerve	1.9	1.0
Inferior gluteal artery (at pelvic exit)	3.1	2.8
Pudendal nerve (at pelvic exit)	2.1	1.3
Rectum (anterolateral wall)	3.2	1.7

## Discussion

The number of POP procedures is expected to increase in the coming decades [12]. Owing to adverse event concerns, transvaginal polypropylene mesh procedures are unlikely to increase in number and the majority of cases will involve native tissue procedures. It is difficult to optimize the surgical outcome of such procedures, and strong and reliable fixation to anatomical landmarks is likely to be the most critical driver of a successful repair. In addition, as a safety requirement the depth of anchor penetration into the ligament should be limited [13, 14]. Several anchoring systems have been developed; however, reliable methods of evaluating these systems are still lacking [3].

In the current study, we demonstrated that the anchors can be safely placed in the correct location, avoiding damage to nerves and vessels in the pelvic cavity, and that these anchors cannot be easily dislocated. Significant variations exist in the thickness of the SSL and the coccygeus muscle. However, this variation is not relevant to the efficacy and safety of the Saffron Fixation System as the anchor will not perforate the complex comprising these two anatomical structures.

With respect to precise anchor placement, clinicians will agree that secure alignment of the fixation device and target is important. The Saffron Fixation Tool distal tip has a flat leading edge and a ridged surface intended to prevent displacement, allowing perpendicular alignment on the ligament, preventing the distal tip from sliding away from the target location during anchor deployment. This has been experienced with the Capio Slim (Boston Scientific), and also with the Caspari suture punch (Arthrotek) that have a blunt rounded head [15, 16]. If the plunger of the Capio Slim or handle of the Caspari suture are activated, the rounded head may slide away from the target tissue. As the Saffron anchor has a small volume and very sharp tip, it is expected to require minimal digital pressure to penetrate the target tissue, which would theoretically minimize the risk of displacement from the target location.

Florian-Rodriguez and colleagues showed that I stitch (AMI) used in patients with a thin SSL could result in damage to the coccygeal branch of the inferior gluteal vessels and nerves [11]. Fixation anchors such as Saffron, should be as small as possible to reduce trauma to the tissue but strong enough to resist intra-abdominal forces. An analysis was carried out to estimate forces for normal apical support under stress. Published articles provided the basis for estimating the relationship between ligament elongation and stress loads [17–19] and it was determined, based on the manufacturer’s design documentation, that a force of 10 N is sufficient to withstand intra-abdominal forces. The data from this study meet this requirement. It remains difficult to compare data obtained from studies performed in frozen cadavers, as variations such as cadaveric tissue quality, freezing technique, thawing conditions, and laboratory temperature exist.

One could argue that the use of frozen non-embalmed cadavers has affected the measured outcomes, as freezing of the body may have affected the performance of the anchoring device. However, the use of multiple anchor deployments, and the fact that the procedure was performed by different surgeons, results in our statement that the observations are generalizable to living subjects. Another point of criticism may be that some surgeons have extensive experience with similar anchoring devices, which could have influenced the objectified efficacy and safety of anchor placement in this study. Also, the fixation device design characteristic preferences of a surgeon may influence the performance in the study setting. We feel that our observations about efficacy and safety of the Saffron Fixation System are valid because the measurements remained consistent among the different surgeons and the different cadavers.

The Saffron Fixation System permits the use of suture and needles of the surgeon’s choice. Comparative studies have not shown superiority of a specific suture when performing SSL fixation [20]. Use of resorbable suture may result in lower complication rates, but their commercial availability remains a challenge. An additional advantage is that as a needleless system, no needles can break and be left in the body during suture placement.

## Conclusion

This innovative anchoring device offers numerous theoretical advantages over existing fixation devices on the market. Future clinical studies need to explore whether the theoretical advantages of this device translate to improved clinical outcomes in comparison with available suturing and anchoring devices.
